# Editorial: Emerging topics in dietary assessment – Edition II

**DOI:** 10.3389/fnut.2022.984915

**Published:** 2022-09-13

**Authors:** Aida Turrini, Laura D'Addezio, Marika Ferrari, Cinzia Le Donne, Lorenza Mistura, Raffaela Piccinelli, Stefania Sette

**Affiliations:** ^1^Independent Researcher (former CREA), Scansano, Italy; ^2^Council for Agricultural Research and Economics (CREA) Research Centre for Food and Nutrition, Rome, Italy

**Keywords:** dietary assessment, dietary surveys, nutritional data, dietary questionnaires, dietary assessment tools, dietary pattern

Quantitative dietary assessment is a cross-cutting theme (1) for several research fields: nutrition, nutritional epidemiology, clinical trials, intervention studies, health and disease, food chain evaluation, consumer behavior, dietary risk assessment, dietary security, and adequacy, as it represents the first step in studies where the diet plays a role. Quantitative evaluation and qualitative understanding allow for identifying food styles to provide a sound scientific information basis for policymakers, educators, citizens, and food system actors (2).

Emerging topics can also be identified following the surveillance and monitoring programs collating secondary data delivered from national statistic bureaus [e.g., (3)]. New indicators can also be proposed to the scientific community. As an example, variables related to the evaluation of diet environmental impact have been recently included in the dietary guidelines and then dietary assessment was also adapted to cope with this issue (4, 5).

The complexity of dietary assessment is high due to its inherent nature, and, overall requires an information system based on several databases like (a) food nomenclatures and coding systems to classify and aggregate foods, and subsequently dietary exposure to contaminants/residues/other harmful substances, (b) recipes to disaggregate into ingredients, (c) portion sizes to quantify the intake, (d) food composition tables to assess energy and nutrients intake, and an (e) adequate software to manage from data entry to data processing, including rules to codify all variables (precision, classes of values) (6). Articles in this Emerging Topics in Dietary Assessment—edition II cover some crucial aspects of this methodological challenge so adding interesting topics further than the previous edition (7).

Complexity deals with determinants and impacts, but also contexts where the surveys are conducted. Particularly, nationwide individual food consumption surveys like those representing the objective of the EU-Menu program undertaken by European Food Safety Authority (EFSA) in 2010 (6). Implementation of Harmonized Food Consumption Data Collection in the Balkan Region according to EFSA EU Menu Methodology Standards describes how a multi-country study can be implemented in a harmonized and standardized way, using a shared platform on “Diet Assess and Plan” (8) that comprises computerized food consumption, anthropometric measurements, and physical activity questionnaires, validated food picture book, and FoodEx2 exposure hierarchy (6) with sets of facet descriptors of the interest. This is part of an initiative in the Capacity Development in Nutrition Research in the Balkan region in the last decade have been toward the creation of contemporary, harmonized Research Infrastructure (RI) compliant with European standards (https://www.esfri.eu/).

Another area continuously evolving concerns the development of a validated food frequency questionnaire (9) suitable for specific cultures, population groups, dietary components, dietary quality, and exposure, and using various technologies either interviewers administered (PAPI, CAPI, CATI, CAWI, CAMI, MAWI) or self-administered (see e.g., https://www.nutritools.org/tools where validated food frequency questionnaire are gathered). PAPI stands for Paper And Pencil Interview, CAPI for Computer-Assisted Personal Interviewing, CATI for Computer-Assisted Telephonic Interviewing, and CAWI for Computer-Assisted Web Interviewing with the variants CAMI—Computer-Assisted Mobile Interviewing, and MAWI—Mobile-Assisted Web Interviewing (10). In the present Research Topic edition, two papers regarding the Food Frequency Questionnaires (FFQ) method have been published: Online Food Frequency Questionnaire From the Cohort of Universities of Minas Gerais (CUME Project, Brazil): Construction, Validity, and Reproducibility and Development of a Food Frequency Questionnaire for Assessing Habitual Intake of Free Sugar Among Children in Saudi Arabia. The first one was an online self-administered questionnaire and the second one was telephonically administered after a web contact with a parent. In both cases, the questionnaires refer to a different population group and a different survey objective. FFQ is a feasible tool for dietary assessment although it is prone to known biases, so validation is recommended, also in the case of a guidance tool like in the Nutritools case (https://www.nutritools.org/login?returnUrl=%2Fquestionnaires) is used.

Economic factors are relevant in evaluating diet sustainability and then influence dietary patterns (11, 12), therefore it is crucial to estimate diet costs, and possibly derive quantities from a household budget survey (HBS) currently carried out by National Statistics Bureaus when other sources are not available (13). Therefore, How to Estimate Food Prices and Diet Costs in Population-Based Studies? is a very relevant question and not easy to address. The lack of price collection within dietary surveys makes it challenging. The paper proposes the approach of pairing features to the households' characteristics in the Health Survey, the HBS data, and the income statistics and using a deflation coefficient from the official consumer price index. An important use of secondary data to fill a gap.

Finally, the quantification issue represents the main task in dietary assessment. It is well-known that all dietary assessment methods are affected by biases (9), so using a standardized measurement method guarantees comparability and hence allows for correctly interpreting the association of explanatory variables to dietary patterns. Using the visual evaluation of food intake is the most used method but it requires the adaptation to different age classes (6) and specific cultures affecting recipes that produce different shaped dishes. In this regard, the present Research Topic includes the Comparison of the Diet Photograph Record to Weighed Dietary Record and 24 h Dietary Recall for Estimating Energy and Nutrient Intakes Among Chinese Preschoolers paper so increasing the number of photographic atlas covering a new population group in China.

Relevant topics are here included, but several others are still far from being addressed, considering the long list of themes that can be included (https://www.frontiersin.org/research-topics/19941/emerging-topics-in-dietary-assessment—edition-ii). So, the conclusion can only be interlocutory, considering a proposal for a permanent Research Topic in which to bring together the news regarding methodological solutions and new tools for measuring individual food consumption in different areas with different devices and different techniques, but also enabling a researcher to have access to the huge number of tools already available maybe considering to build a wiki space providing the necessary information and tools.

## Author contributions

AT has written the first draft. LD'A, MF, CL, LM, RP, and SS have revised, discussed, and modified the text. All authors have agreed on the content. All authors contributed to the article and approved the submitted version.

## Conflict of interest

The authors declare that the research was conducted in the absence of any commercial or financial relationships that could be construed as a potential conflict of interest.

## Publisher's note

All claims expressed in this article are solely those of the authors and do not necessarily represent those of their affiliated organizations, or those of the publisher, the editors and the reviewers. Any product that may be evaluated in this article, or claim that may be made by its manufacturer, is not guaranteed or endorsed by the publisher.

## References

[1] Turrini A. Perspectives of dietary assessment in human health and disease. Nutrients. (2022) 14:830. 10.3390/nu14040830 35215478 PMC8877528

[2] Fanzo J Haddad L Schneider KR Béné C Covic NM Guarin A. Viewpoint: rigorous monitoring is necessary to guide food system transformation in the countdown to the 2030 global goals. Food Policy. (2021) 104:20. 10.1016/j.foodpol.2021.102163

[3] Elmadfa I (editor). European Nutrition and Health Report 2009. Forum Nutr. Basel, Karger (2009) 62:250–390. 10.1159/000242376. Available online at: https://www.karger.com/Article/Abstract/242376 (accessed September 4, 2022).20104000

[4] Ahmed S Downs S Fanzo J. Advancing an integrative framework to evaluate sustainability in national dietary guidelines. Front Sustain Food Syst. (2019) 3:76. 10.3389/fsufs.2019.00076

[5] Rossi L Berni Canani S Censi L Gennaro L Leclercq C Scognamiglio U. The 2018 revision of Italian dietary guidelines: development process, novelties, main recommendations, and policy implications. Front Nutr. (2022) 9:861526. 10.3389/fnut.2022.861526 35399680 PMC8990302

[6] EFSA- European Food Safety Authority. Guidance on the EU Menu methodology. EFSA J. (2014) 12:3944. 10.2903/j.efsa.2014.394432704313

[7] Turrini A D'Addezio L Dhurandhar E Ferrari M Le Donne C Mistura L. Editorial: emerging topics in dietary assessment. Front Nutr. (2019) 19:176. 10.3389/fnut.2019.00176 31803751 PMC6877603

[8] Gurinović M Milešević J Kadvan A Nikolić M Zeković M Djekić-Ivanković M. Development, features and application of DIET ASSESS & PLAN (DAP) software in supporting public health nutrition research in Central Eastern European Countries (CEEC). Food Chem. (2018) 238:186–94. 10.1016/j.foodchem.2016.09.114 28867092

[9] Thompson FE Subar AF. Dietary assessment methodology. In: Nutrition in the Prevention and treatment of Diseases Chapter 1. Bethesda, MD: National Cancer Institute (2013). 5-48. Available online at: https://epi.grants.cancer.gov/dietary-assessment/Chapter%201_Coulston.pdf

[10] Kagerbauer M Manz W Zumkeller D. Analysis of PAPI, CATI, and CAWI methods for a multiday household travel survey. In: Zmud J Lee-Gosselin M Munizaga M Carrasco JA editor. Transport Survey Methods. Bingley: Emerald Group Publishing Limited (2013). 289–304.

[11] Seubelt N Michalke A Gaugler T. Influencing factors for sustainable dietary transformation— A case study of german food consumption. Foods. (2022) 11:227. 10.3390/foods11020227 35053959 PMC8775007

[12] Springmann M Wiebe K. Mason-D'Croz D, Sulser TB, Rayner M, Scarborough P. Health and nutritional aspects of sustainable diet strategies and their association with environmental impacts: a global modelling analysis with country-level detail. Lancet Planet Health. (2018) 2:e451–61. 10.1016/S2542-5196(18)30206-7 30318102 PMC6182055

[13] Naska A Berg M-A Cuadrado C Freisling H Gedrich K Gregorič M. Food balance sheet and household budget survey dietary data and mortality patterns in Europe. Br J Nutr. (2009) 102:166–71. 10.1017/S000711450809466X 18986595

